# Primary Afferent-Derived BDNF Contributes Minimally to the Processing of Pain and Itch

**DOI:** 10.1523/ENEURO.0402-18.2018

**Published:** 2018-12-26

**Authors:** Todd Dembo, João M. Braz, Katherine A. Hamel, Julia A. Kuhn, Allan I. Basbaum

**Affiliations:** 1 Department of Anatomy, University of California, San Francisco, San Francisco, CA 94158

**Keywords:** BDNF, dorsal root ganglia, itch, neuropathic pain, spinal cord, transgenic knock-out

## Abstract

BDNF is a critical contributor to neuronal growth, development, learning, and memory. Although extensively studied in the brain, BDNF is also expressed by primary afferent sensory neurons in the peripheral nervous system. Unfortunately, anatomical and functional studies of primary afferent-derived BDNF have been limited by the availability of appropriate molecular tools. Here, we used targeted, inducible molecular approaches to characterize the expression pattern of primary afferent BDNF and the extent to which it contributes to a variety of pain and itch behaviors. Using a *BDNF-LacZ* reporter mouse, we found that BDNF is expressed primarily by myelinated primary afferents and has limited overlap with the major peptidergic and non-peptidergic subclasses of nociceptors and pruritoceptors. We also observed extensive neuronal, but not glial, expression in the spinal cord dorsal horn. In addition, because BDNF null mice are not viable and even Cre-mediated deletion of BDNF from sensory neurons could have developmental consequences, here we deleted BDNF selectively from sensory neurons, in the adult, using an advillin-Cre-ER line crossed to floxed BDNF mice. We found that BDNF deletion in the adult altered few itch or acute and chronic pain behaviors, beyond sexually dimorphic phenotypes in the tail immersion, histamine, and formalin tests. Based on the anatomical distribution of sensory neuron-derived BDNF and its limited contribution to pain and itch processing, we suggest that future studies of primary afferent-derived BDNF should examine behaviors evoked by activation of myelinated primary afferents.

## Significance Statement

The neurotrophin BDNF is a critical contributor to neuronal growth, development, and synaptic plasticity, and its expression in primary sensory neurons has been implicated in pain processing. However, there is little consensus as to the sensory neuron subtypes that express BDNF or whether sensory neuron-derived BDNF facilitates or inhibits pain generation. Here, we used a BDNF reporter mouse and demonstrate that BDNF predominates in myelinated sensory neurons and is expressed in many spinal cord dorsal horn neurons. In addition, in studies in which BDNF was deleted, in the adult, from all sensory neurons, we found limited deficits in pain or itch processing.

## Introduction

The neurotrophin BDNF is a critical contributor to neuronal growth and development, synaptic transmission, learning, and memory (Binder and Scharfman, 2004). Although widely synthesized in the CNS (Conner et al., 1997), BDNF is also expressed in primary sensory neurons of the dorsal root ganglion (DRG; Barakat-Walter, 1996) and trigeminal ganglion (TG; Wetmore and Olson, 1995). Several studies reported that BDNF is expressed in a population of small to medium-diameter DRG neurons (Michael et al., 1997; Thompson et al., 1999; Heppenstall and Lewin, 2001; Luo et al., 2001; Obata et al., 2006; Salio and Ferrini, 2016) and its expression pattern changes after nerve or tissue damage (Cho et al., 1997; Zhou et al., 1999; Fukuoka et al., 2001; Obata et al., 2006; Lin et al., 2011). For example, tissue injury increases the number of BDNF-expressing neurons in the DRG, with *de novo* expression in non-nociceptive, large-diameter cells and decreased expression in the small-diameter, nociceptive population. On the other hand, another report emphasized the extensive expression in presumptive proprioceptive afferents, rather than nociceptors (Betley et al., 2009). Conceivably some of the lack of agreement reflects a potential caveat of the anatomic studies, namely that because the BDNF null mutation is generally lethal (Ernfors et al., 1994) studies that characterized BDNF expression likely lacked appropriate controls for antibody and probe specificity.

The majority of studies indicate that BDNF is pro-nociceptive. For example, subcutaneous injection of BDNF modestly decreased heat pain threshold (Thompson et al., 1999). Intrathecal injections of BDNF or a TrkB (BDNF receptor) scavenger also decreased heat (Shu et al., 1999; Groth and Aanonsen, 2002) and mechanical thresholds (Groth and Aanonsen, 2002; Coull et al., 2005; Bao et al., 2014). Very recently (Dhandapani et al., 2018) demonstrated that TrkB-expressing sensory neurons are myelinated, low-threshold mechanoreceptors that transmit innocuous touch and their ligand-targeted photoablation reduces mechanical allodynia in a wide variety of neuropathic pain models in the mouse. Similarly, inhibiting BDNF signaling with an intrathecal antibody decreased thermal hyperalgesia in the spinal nerve ligation model of neuropathic pain (Fukuoka et al., 2001).

Taking a different approach (Zhao et al., 2006) selectively deleted BDNF from the NaV1.8 subset of sensory neurons and observed reduced nocifensive behavior after carrageenan-induced inflammation and in the second phase of the formalin test. Paradoxically, these mice were heat hypersensitive, suggesting that BDNF is antinociceptive, a result consistent with a report demonstrating GABA-mediated inhibitory effects of intrathecal BDNF (Pezet et al., 2002). In the same study, Zhao et al. (2006) found no effect on nerve injury-induced mechanical hypersensitivity. Surprisingly, the same group found opposite results, namely decreased responsiveness in a test of heat pain and reduced nerve injury generated mechanical allodynia when they deleted BDNF from sensory neurons in the adult (Sikandar et al., 2018). To address the lack of consensus, here we provide a comprehensive analysis of BDNF expression in sensory neurons and spinal cord and reexamined the effect of its selective deletion from sensory neurons on both pain and itch processing. Importantly, because BDNF signaling is sexually dimorphic (Liu et al., 2012), we studied both male and female adult mice. We report that BDNF is predominantly expressed by myelinated afferents and contributes minimally to pain and itch.

## Materials and Methods

### Animals

Animal experiments were approved by the University of California, San Francisco Institutional Animal Care and Use Committee and were conducted in accordance with the National Institutes of Health Guide for the Care and Use of Laboratory animals. *Bdnf ^fl/fl^* and Ai14 (tdTomato) reporter mice were purchased from The Jackson Laboratory. Crossing these mice resulted in double transgenic mice that provided an anatomic fate map of the expression of BDNF. *Bdnf ^LacZ/+^* reporter mice were kindly provided by Dr. Zachary Knight at University of California, San Francisco and Dr. Kevin Jones (Colorado; Gorski et al., 2003). This transgenic mouse expresses a knock-in floxed BDNF followed by a lacZ expression cassette. Crossing this mouse with an Advillin Cre-ERT2 line resulted in knock-out of BDNF and expression of lacZ at the BDNF locus, which leads to lacZ expression in cells that normally express BDNF in the adult. This mouse line was used for both anatomic analyses of BDNF expression (by following lacZ) and behavioral analyses of BDNF mutant mice following tamoxifen administration. The Advillin-CreERT2 mice were kindly provided by Dr. Ardem Patapoutian at the Scripps Institute.

### Tamoxifen-induced recombination

Tamoxifen (Sigma) was dissolved in corn oil by vigorous vortexing for 30 min. Cre recombination was induced by injecting animals with tamoxifen (150 mg/kg, i.p.) for five consecutive days, as described previously (Lau et al., 2011). Vehicle-treated control mice were injected with corn oil instead of tamoxifen.

### Behavioral analyses

For all behavioral tests, animals were first habituated for 1 h in Plexiglas cylinders. The experimenter was always blind to whether the animal received tamoxifen. All mechanical (Von Frey), thermal (Hargreaves, tail immersion, hotplate, acetone), and ambulatory (rotarod) tests were conducted two weeks after the last tamoxifen injection as described previously (Cao et al., 1998; Wang et al., 2013). For the capsaicin test, capsaicin (Sigma) was first dissolved in 100% ethanol (3.0 mg/ml). This stock was mixed 1:1:8 with Tween 80 (Sigma) and saline, and injected into the hindpaw (10 μl), with behavior then video recorded for the next 5 min. Behavior was scored as the total duration of hindpaw licking. For the acetone test, behavior was quantified as the total duration of hindpaw lifting, licking or shaking over five consecutive, 30-s trials.

### Pruritogen-evoked scratching

At least 24 h before testing, mice were shaved at the nape of the neck under isoflurane anesthesia. The following pruritogens were dissolved in saline and injected (50 μl, s.c.) into the neck: chloroquine (Sigma, 100 μg), endothelin-1 (Sigma, 25 ng), histamine (Sigma 500 μg), SLIGRL (Sigma, 100 μg), and thymic stromal lymphopoietin (TSLP; Sigma, 2.5 μg). The mice were video recorded, and from these videos, we counted the total number of discrete scratching bouts that occurred during the first 30 min after injection.

### Complete Freund’s adjuvant (CFA)

The CFA model of chronic inflammation was induced as described previously (Cao et al., 1998). Briefly, CFA (Sigma) was diluted 1:1 with saline and vortexed for 30 min. When fully suspended, we injected 20 μl of CFA into one hindpaw. Heat and mechanical withdrawal thresholds were measured before the injection (baseline) and 3 d after using the Hargreaves and Von Frey tests, respectively.

### Formalin test

The formalin test was conducted as described previously (Shields et al., 2010). Briefly, formalin was injected into the plantar surface of the hindpaw (10 μl, 2% in saline) and nocifensive behavior was video recorded over the following hour. The cumulative duration of all behaviors (licking, lifting, shaking, biting) was measured in 5-min bins.

To study formalin-induced Fos expression, animals were perfused 1.5–2 h after the formalin injection, or 48 h after injection to immunostain for ATF3 in the DRG. See below, Immunohistochemistry, for the list of antibodies and concentrations used. In some animals, we also studied post-formalin-induced mechanical hypersensitivity (allodynia), 1 d after the formalin injection (Zeitz et al., 2004).

### Paclitaxel model of chemotherapy-induced neuropathic pain

Paclitaxel (Sigma) was diluted in Kolliphor EL (Sigma, 1:1 with 100% ethanol) to a final concentration of 6.0 mg/ml. Aliquots of 20 μl were stored at –20°C until use, when they were diluted 15× with saline. Mice were injected intraperitoneally with 4.0 mg/kg, once per day, for five consecutive days. Heat and mechanical thresholds were measured at 12 and 13 d, respectively, after the final injection.

### Spared-nerve injury (SNI) model of neuropathic pain

The SNI model was produced as described previously (Shields et al., 2003). Briefly, under isofluroane anesthesia, two of the three branches of the sciatic nerve were ligated and transected distally, leaving the tibial nerve intact. Behavior was tested 5, 7, and 14 d after injury, and immunohistochemistry was performed one week after injury.

### Immunohistochemistry

Tissue was immunostained as described previously (Bráz et al., 2012). Antibodies used included ATF3 (rabbit, 1:2 k, Santa Cruz Biotechnology), Fos (rabbit, 1:5 k, Oncogene), Iba1 (rabbit, 1:1 k, Wako), NeuN (mouse, 1:5 k, Sigma), β-Gal (chicken, 1:10 k, Abcam), TRPV1 (guinea pig, 1:5 k, generous gift of the David Julius lab), NF200 (mouse, 1:20 k, Sigma), CGRP (mouse, 1:10 k, Sigma), tyrosine hydroxylase (rabbit, 1:5 k, Millipore), and biotinylated IB4 (goat, 1:500, Vector Labs). Fluorescent secondary antibodies were used at a 1:1 k dilution; streptavidin-conjugated fluorophore was used at 1:5 k.

### *In situ* hybridization

*In situ* hybridization was performed using fresh DRG tissue from adult mice (8–10-week-old), following Advanced Cell Diagnostics’ protocol. DRG tissues were dissected out, instantaneously frozen on dry ice, and kept at –80°C until use. DRG cryostat sections (12 µm) were fixed at 4°C in 4% formaldehyde for 15 min, washed twice in PBS, and dehydrated through successive ethanol steps (50%, 70%, and 100%) for 5 min each and dried at room temperature. After a 30-min incubation step with protease IV, sections were washed twice in PBS and incubated at 40°C with RNAscope-labeled probes (TRPM8, BDNF, or CGRP) for 2 h in a humidified chamber. Sections were then washed twice in washing buffer and incubated with four successive “signal amplifying” solutions at 40°C, for 15–30 min each. After two washes, sections were dried and covered with mounting media containing DAPI.

### BDNF ELISA

Advillin-CreERT2:*Bdnf ^fl/fl^* mice were injected with tamoxifen or vehicle (corn oil) as described above, in the section Tamoxifen-induced recombination. Two weeks after the final injection, spinal cords (5.0 mm of lumbar enlargement) and TGs were homogenized in 20× volume lysis buffer (pH 7.0) containing Tris-HCl (100 mM), NaCl (1.0 M), EDTA-Na_2_ (4 mM), bovine serum albumin (2%), Triton X-100 (2%), sodium azide (0.1%), and complete ULTRA protease inhibitor cocktail (Roche). Homogenates were centrifuged at 14 k × *g* for 30 min at 4°C. Spinal cord supernatants were diluted 10× and TG supernatants diluted 5×. All supernatants were immediately assayed with the Human BDNF SimpleStep ELISA kit (Abcam). Absorbance was measured at 450 nm with a Biotek H4 Plate Reader.

### Quantitative PCR

Tissue was homogenized in TRIzol (Ambion) and RNA was purified using the PureLink RNA Mini kit with on-column DNase treatment (Ambion). cDNA was prepared with the SuperScript III First-Strand Synthesis SuperMix for qRT-PCR (Invitrogen). mRNA levels were quantified with the Bio-Rad CFX Connect System using PowerUp SYBR Green Master Mix (Applied Biosystems). All transcripts were normalized to actin.

### Genotyping

All genotyping parameters followed the instructions listed on The Jackson Laboratory website. The primer sets used included *BDNF^LacZ/+^*: GGTCTGAAATTACAAGCA GATGG and TGTCCGTGGACGTTTACTTCT; Advillin-CreERT2: GCGGTCTGGCAGTAAAAACTATC and GTGAAACAGCATTGCTGTCACTT; *BDNF ^fl/fl^*: TGTGATTGTGTTTCTGGTGAC and GCCTTCATGCAACCGAAGTATG.

### Imaging

All images were taken on an LSM 700 confocal microscope (Zeiss) equipped with 405-nm (5-mW fiber output), 488-nm (10-mW fiber output), 555-nm (10-mW fiber output), and 639-nm (5-mW fiber output) diode lasers using a 20× Plan-Apochromat (20×/0.8) objective (Zeiss). Image acquisition was done with ZEN 2010 (Zeiss). Image dimensions were 1024 × 1024 pixels with an image depth of 12 bits. Two-times averaging was applied during image acquisition. Laser power and gain were adjusted to avoid saturation of single pixels. Adjustment of brightness/contrast and changing of artificial colors (LUT) were done in Fiji/ImageJ. The same imaging parameters and adjustments were used for all images within an experiment.

### Experimental design and statistical analysis

For cell counting of DRG neurons, we collected 12-μm cryosections of L4/5 DRGs from at least three animals per group. The sections were directly mounted on Superfrost microslides. To avoid double counting of the same cell, we mounted, immunostained, and counted neurons in every fifth section of each ganglion. ATF3-immunoreactive neurons were identified using the particle analyzer function in ImageJ. To quantify the percentage of β-Gal-immunoreactive (BDNF-expressing) DRG neurons that coexpressed NF200, peripherin, CGRP, TRPV1, IB4, or TH, we counted at least 100 β-Gal-positive neurons for each mouse and calculated the percentage of double-labeled neurons (β-Gal/neuronal marker).

All behavioral studies were performed blind to treatment (e.g., tamoxifen vs corn oil). Mice were randomly assigned to experimental groups with eight to nine mice per group, a number based on our previous and other studies using comparable tests. Importantly, in the different behavioral experiments, the mice served as their own controls. Data were collected before and after tamoxifen-induced deletion of BDNF. In one experiment, post-formalin-induced mechanical hypersensitivity, only six mice per group were studied. When results from male and female mice did not differ, the results were combined.

### Statistical analyses

All statistical analyses were performed with Prism (GraphPad). Anatomic and behavioral data are reported as mean ± SEM. In all experiments where males and females were compared, two-way ANOVA was used first (with Bonferroni correction for multiple comparisons) to determine whether any of the mean endpoints were significantly different between sex and genotype. If differences were found, males and females were compared separately in the final analysis using two-way ANOVA with Bonferroni correction. If no differences were found, male and female data were grouped together and the pooled endpoints were compared using *t* tests or ANOVA. Specifically, if pooled endpoints were independent from one another (e.g., when comparing the effects of different pruritogens or looking at phases of the formalin test), multiple *t* tests were used with Bonferroni correction for multiple comparisons. When endpoints were not independent (e.g., in the hotplate test), ANOVA was used with Bonferroni correction for multiple comparisons.

## Results

### Anatomic characterization of *BDNF^LacZ/+^* mice

Previous attempts to characterize the expression of BDNF in primary afferent neurons relied on immunohistochemistry or *in situ* hybridization. However, BDNF antibodies are known to be problematic, and *in situ* studies, although more reliable, were not comprehensive. Because of the importance of relating expression patterns to sensory neuron subpopulations, here we took advantage of a knock-in reporter animal that expresses β-Gal under control of the BDNF promoter. This *BDNF^LacZ/+^* animal has previously been used to characterize BDNF expression in the brain (Xu et al., 2003), but to our knowledge, this is the first analysis in sensory neurons or spinal cord.

**Figure 1.**
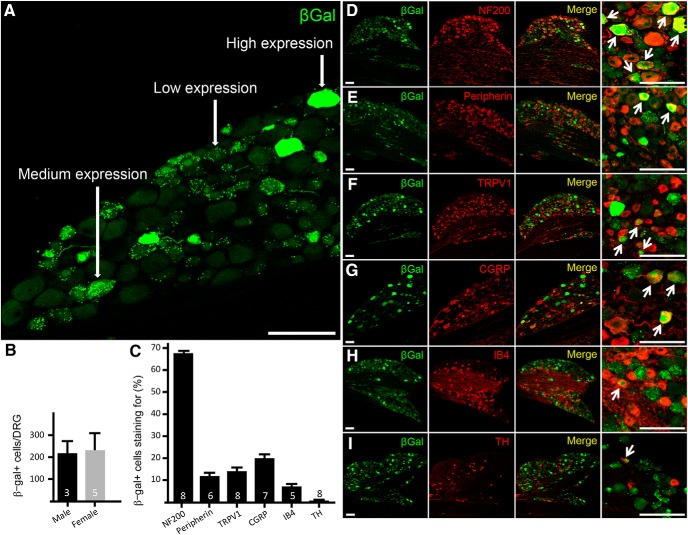
BDNF expression in the DRG of the *BDNF-LacZ* knock-in reporter mouse. ***A***, β-Gal expression was found in a heterogeneous population of primary afferent neurons. Levels of expression varied, with some cells completely filled by β-Gal (high expression), others with punctate nuclear and cytoplasmic staining (medium expression), and some with only punctate cytoplasmic staining (low expression). ***B***, The number of β-Gal-positive DRG cells did not differ in male and female mice. ***C***, By counterstaining with a panel of markers of subpopulations of DRG neurons, we determined that ∼67% of BDNF-expressing neurons express NF200 (***D***), a marker of myelinated fibers. Only ∼12% of BDNF cells expressed peripherin (***E***), a marker of most small-diameter afferents. Similarly, only ∼14% of BDNF-positive afferent expressed TRPV1 (***F***) and ∼20% expressed CGRP (***G***), markers of small-diameter, peptidergic nociceptors. ***H***, IB4, a lectin that binds small, non-peptidergic nociceptors, was only found in ∼10% of BDNF neurons. Finally, there was virtually no overlap (∼0.5%) with TH (***I***), a marker of small-diameter, low-threshold, mechanosensory afferents. Data are mean ± SEM. Statistical significance was determined by Student’s *t* test. Arrows indicate cells with overlap of β-Gal with other markers. Numbers of animals are indicated in ***B***, ***C***. Scale bars = 100 μm.

Our investigation focused on DRGs L4/5, as these levels innervate the plantar surface of the hindpaw, where most of our behavioral experiments were conducted. Figure 1 illustrates that the intensity of β-Gal immunoreactivity within DRG neurons varied considerably. Immunolabeling in high-expressing cells (∼32.3 ± 4.5% of total BDNF+ neurons; *N* = 5) completely filled both the nucleus and cytoplasm. Medium-expressing cells were characterized by punctate staining of the nucleus and cytoplasm. Low-expressing cells had punctate staining in the cytoplasm and little to no nuclear labeling (medium-to-low expressing cells: ∼67.8 ± 4.6% of total BDNF+ neurons; *N* = 5; Fig. 1*A*). For the purposes of this investigation, all β-Gal-positive cells were treated equally, regardless of staining intensity. Importantly, because the β-Gal reporter animal is not a fusion construct between LacZ and BDNF, the β-Gal pattern defines the neurons that express BDNF, rather than the intracellular staining pattern of BDNF itself. Cell counts showed that there are ∼200 β-Gal-positive neurons per DRG, with similar numbers in male and female mice (Fig. 1*B*).

**Figure 2.**
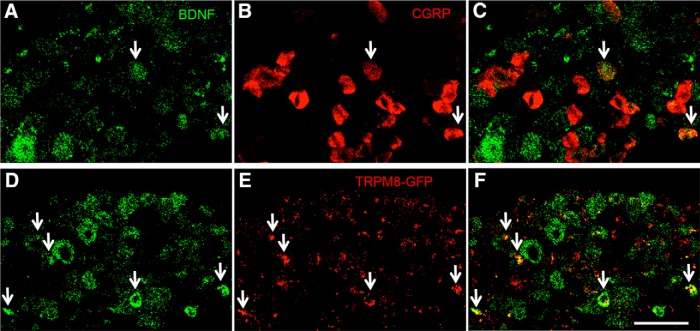
Coexpression of BDNF with the neuropeptide CGRP or the cold receptor, TRPM8. ***A***, Double *in situ* hybridization confirmed that some BDNF mRNA-positive neurons (green in ***A***, ***D***), coexpress CGRP (red in ***B***, ***C***). ***D–F***, Double *in situ* hybridization for GFP (red in ***E***, ***F***) and BDNF (green in ***D***) in DRG neurons from TRPM8-GFP knock-in mice show that ∼23% of BDNF-positive neurons coexpress TRPM8. Scale bar = 100 μm.

We next characterized β-Gal-positive cells with a panel of markers that distinguish subpopulations of DRG neurons (Fig. 1*C–I*). The majority of the β-Gal neurons (∼67%) coexpressed NF200 (Fig. 1*D*), a marker of myelinated afferents; ∼12% expressed peripherin, a marker of most unmyelinated C fibers (Fig. 1*E*). The relative abundance in myelinated afferents is consistent with an earlier report, using a *BDNF-LacZ* knock-in mouse, that at least 80% of proprioceptive neurons express BDNF (Betley et al., 2009). A small percentage of the BDNF neurons coexpressed TRPV1 (∼14%; Fig. 1*F*) or CGRP (∼20%; Figs. 1*G*, 2*B–D* ), markers of small, peptidergic nociceptive fibers and some A δ afferents. Only ∼7% of the lacZ-positive neurons bound IB4 (Fig. 1*H*), which marks small, non-peptidergic, nociceptive afferents. Finally, we rarely detected β-Gal-positive neurons that coexpressed tyrosine hydroxylase (TH; ∼0.5%; Fig. 1*I*), a marker of small, unmyelinated, low-threshold mechanoreceptive afferents (CLTMs). By *in situ* hybridization, we found that ∼23% of the BDNF mRNA-expressing sensory neurons coexpressed the menthol receptor TRPM8 (Fig. 2), a marker of small, cold-responsive cells. These cells are largely non-overlapping with those that express peripherin, TRPV1, CGRP, or IB4 (Dhaka et al., 2008; Takashima et al., 2010).

**Figure 3.**
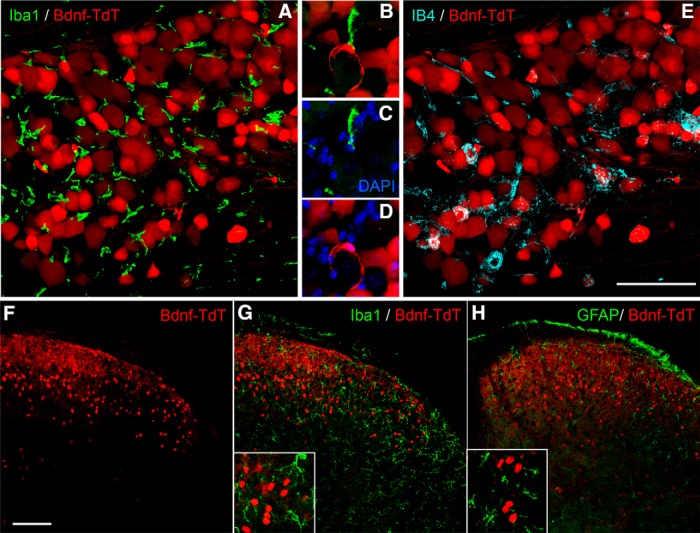
Fate map of BDNF expression in sensory ganglia and spinal cord neurons ***A–E***, In double transgenic BDNF-Cre/floxed-tdTomato mice, tdTomato (red) was recorded in a large number of DRG neurons, but not in macrophages, identified by expression of Iba1 (green in ***A***, ***B***). A wide variety of DRG neurons expressed tdTomato, some of which were marked by IB4 binding (blue in ***E***). Insets ***B–D*** show high magnifications of cells from ***A***. ***F–H***, TdTomato (red) was also expressed in spinal cord neurons, but not in microglia (Iba1+, green in ***G***) or astrocytes (GFAP+, green in ***H***). Insets show high magnification of labeled cells. Scale bars = 100 μm.

**Figure 4.**
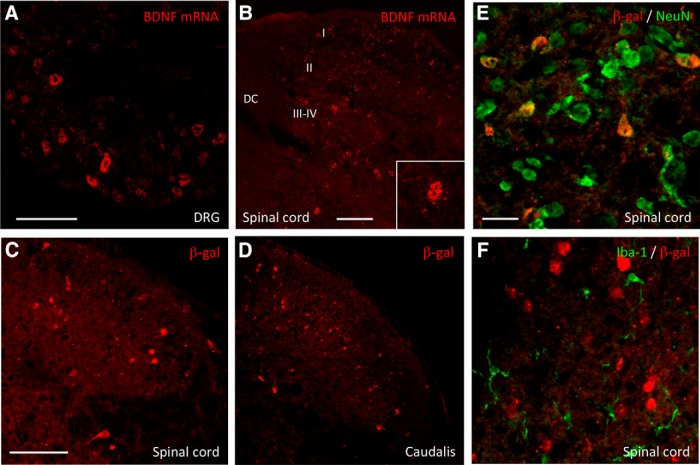
BDNF expression in sensory ganglia and spinal cord neurons of the adult mouse. ***A***, ***B***, mRNA expression of BDNF in adult DRG (***A***) and spinal cord (***B***) neurons revealed by *in situ* hybridization. BDNF mRNA was detected in all laminae of the dorsal horn. ***C–F***, In the adult *BDNF-LacZ* knock-in reporter mice, β-Gal expression was also found in spinal cord (***C***) and trigeminal nucleus caudalis neurons (***D***). We only recorded β-Gal expression in neurons (NeuN+, green in ***E***), not in microglia (Iba1+, green in ***F***). Scale bars = 100 μm (***A***, ***B***, ***D–F***) and 25 μm (***C***). DC: dorsal column; I, II, III–IV: laminae I, II and III–IV.

In parallel studies, we crossed mice that express Cre recombinase under control of the BDNF promoter, with Ai14 (tdTomato-expressing) reporter mice. Compared to the number of β-Gal-positive neurons in the adult, the tdTomato expression showed that BDNF is expressed in a much larger number of sensory neurons (Fig. 3*A–E*). This finding reflects the fact that this approach provides a fate map of all neurons that express BDNF, from the embryo through the adult. We also recorded a large number of spinal cord neurons in dorsal horn laminae I-V (Fig. 3*F–H*). On the other hand, we never found BDNF in non-neuronal cells in either the DRG (e.g., satellite cells or macrophages; Fig. 3*A*,*B*) or in the spinal cord, e.g., microglia (Fig. 3*G*) and astrocytes (Fig. 3*H*). Because the tdTomato fate map in spinal cord neurons may have resulted only from embryonic Cre recombination, we also examined BDNF expression in adult spinal cord by *in situ* hybridization. Figure 4 illustrates that BDNF is indeed expressed in spinal cord neurons in adult mice.

### Conditional, primary afferent-driven deletion of BDNF

**Figure 5.**
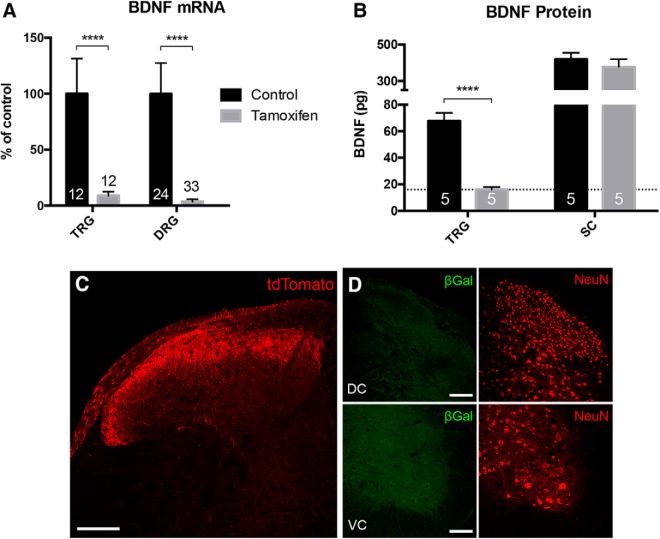
Advillin-CreERT2-mediated primary afferent deletion of BDNF. ***A***, Compared to control animals treated with vehicle, qRT-PCR of DRG and trigeminal ganglia (TRG) showed highly significant depletion of BDNF (∼91% and ∼97%, respectively) after tamoxifen-induced Cre recombination. ***B***, ELISA showed that BDNF peptide was reduced in the TRG, but not spinal cord (SC). The dotted line indicates the detection limit of the ELISA; values below this line were statistically indistinguishable from zero. ***C***, Consistent with the qPCR and ELISA findings, after crossing Advillin-CreERT2 animals with a tdTomato reporter line and treating with tamoxifen, we only detected tdTomato in presumptive primary afferent terminals of the spinal cord dorsal horn. ***D***, Furthermore, double transgenic Advillin-CreERT2/floxed-LacZ mice did not exhibit any β-Gal staining in the spinal cord after tamoxifen. Together, these results indicate that Advillin-CreERT2 does not drive Cre expression in the spinal cord, the neurons of which are indicated by NeuN immunostaining (red in ***D***) and that tamoxifen-induced recombination is selective for sensory ganglia. Data are mean ± SEM. Statistical significance was determined by multiple *t* tests with Bonferroni correction for multiple comparisons; **p < 0.01, ****p < 0.0001. Numbers of animals are indicated in ***A***, ***B***. Scale bars = 100 μm. DC: dorsal cord; VC: ventral cord.

To address concerns about the effect of developmental BDNF deletion (i.e., using a Cre line that expresses in the embryo), here we took advantage of an inducible Advillin-CreERT2, which is expressed in >90% all primary afferents (Lau et al., 2011). Crossing these mice with a floxed-BDNF line and treating adult mice with tamoxifen, generates a conditional knock-out (cKO) mouse. Although a previous report using this mouse found no difference in the number of neurons in the DRG after deletion (Zhao et al., 2006), those authors did not quantify the magnitude of the BDNF deletion in the DRG. Here, using qPCR, we found there was an almost complete deletion of BDNF mRNA from DRG and TG neurons (∼96% and ∼91%, respectively; Fig. 5*A*). A BDNF ELISA further demonstrated that BDNF protein levels in the TG dropped below the detection limit of the assay (Fig. 5*B*). Importantly, BDNF protein levels were unchanged in the spinal cord, which indicates, as expected, that Advillin-CreERT2 does not drive recombination in spinal cord neurons. This conclusion was confirmed in the Advillin-CreERT2-dependent tdTomato reporter animals, in which we only detected tdTomato in presumptive primary afferent terminals in spinal dorsal horn (Fig. 5*C*). We conclude that the BDNF knock-out was restricted to primary afferent sensory neurons.

### Baseline tests of pain and itch processing

**Figure 6.**
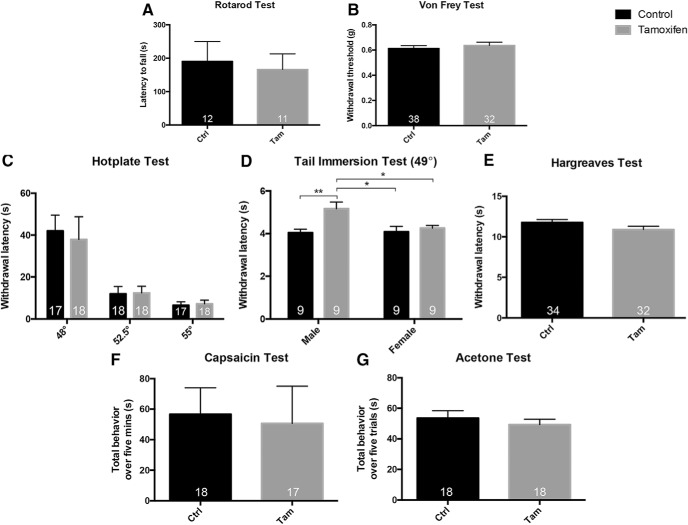
Baseline nociceptive responsiveness of the BDNF-cKO mice ***A***, ***B***, BDNF cKO mice (Tam; gray) displayed normal behavior compared to vehicle-injected controls (Ctrl) on the rotarod test (***A***) of motor behavior and Von Frey test (***B***) of mechanical thresholds. ***C***, Tamoxifen-treated cKO mice also responded normally on the hotplate test at all tested temperatures. ***D***, Male knock-out mice showed a significant hyposensitivity in the tail immersion test compared to all other groups. As all knock-out mice responded normally in the Hargreaves (***E***), capsaicin (***F***), and acetone (***G***) tests results from male and female were combined. Numbers of animals are indicated on graphs. Data are mean ± SEM. For ***A***, ***B***, ***E–G***, statistical significance was determined by Student’s *t* test. For ***C***, statistical significance was determined by ANOVA with Bonferroni correction for multiple comparisons. For ***D***, statistical significance was determined by two-way ANOVA with Bonferroni correction for multiple comparisons; *p < 0.05, **p < 0.01.

**Figure 7.**
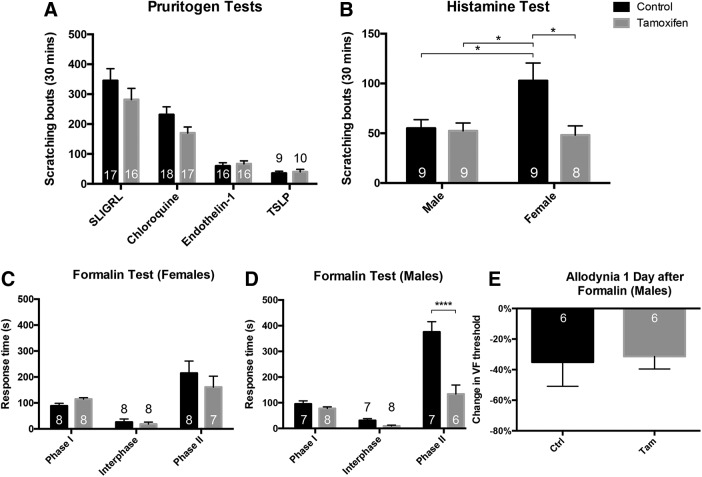
Pruritogen and formalin responsiveness in BDNF-cKO mice. ***A***, Tamoxifen-treated cKO mice responded normally to injections of acute pruritogens including SLIGRL, chloroquine, endothelin-1, and TSLP. ***B***, Female mice showed statistically elevated responses to histamine compared to vehicle-injected control animals, but the increase disappeared after tamoxifen, i.e., after BDNF deletion. ***C***, Female tamoxifen-treated cKO mice did not respond differently from corn oil-injected controls during any of the three phases of the formalin test**. *D***, Male BDNF cKO mice showed a large, highly significant decrease in their responses, but only during the second phase of the formalin test. ***E***, Despite the dramatic reduction in behavior during the second phase of the formalin test, male cKO mice nevertheless developed significant mechanical allodynia 24 h after the formalin injection equivalent to that recorded in the control mice. Data are mean ± SEM. For ***A***, ***C***, ***D***, statistical significance was determined by multiple *t* tests with Bonferroni correction for multiple comparisons. For ***B***, statistical significance was determined by two-way ANOVA with Bonferroni correction for multiple comparisons. For ***E***, statistical significance was determined by Student’s *t* test; *p < 0.05, ****p < 0.0001. Numbers of animals are indicated on graphs.

The conditional BDNF knock-out (cKO) mice responded normally on the rotarod test (Fig. 6*A*), which demonstrates that there were no significant motor abnormalities that could compromise tests that assayed hindlimb withdrawal. This finding also suggests that the expression of BDNF in proprioceptive afferents (Betley et al., 2009) is not required for normal proprioception. Mice also responded normally in the Von Frey test, which measures mechanical sensitivity (Fig. 6*B*). BDNF cKO and vehicle-treated control mice also did not differ in nociceptive tests of thermal sensation, including the hotplate (Fig. 6*C*) and Hargreaves tests (Fig. 6*E*) for heat, and the acetone test of cold sensitivity (Fig. 6*G*). Chemonociception, in response to hindpaw injection of capsaicin, was also unchanged in the cKO mice (Fig. 6*F*). Surprisingly, the BDNF cKO mice showed a sexually dimorphic phenotype in the tail immersion test of heat sensitivity at 49°C; male, but not female, knock-out mice had increased withdrawal latencies (Fig. 6*D*). Finally, scratching in response to most of the tested pruritogens did not differ (Fig. 7*A*) between wild-type and cKO mice, with the exception of histamine (Fig. 7*B*), where we found that tamoxifen treatment (i.e., BDNF deletion) significantly reduced histamine-induced scratching, but only in female mice. However, as baseline scratching was significantly greater in female than male mice, the BDNF deletion actually normalized responsiveness in the female mice.

### Sexually dimorphic, reduced responsiveness in the formalin test of inflammatory pain

The formalin test is a model of persistent inflammation and considered to be a reliable correlate of tissue injury-induced pain. As noted above the previous studies (using NaV1.8-Cre or advillin-Cre-ER-mediated deletion of BDNF) found reduced responsiveness in the second phase of the formalin test, which lasts from ∼15–50 min after the formalin injection (Zhao et al., 2006). Interestingly, we found that male, but not female mice, showed dramatic reductions in their responses during the second phase of the test (Fig. 7*C*,*D*). This result was unexpected as the first (NaV1.8) study of Zhao et al. (2006) only used female mice and recorded decreased second phase formalin behavior. As their subsequent study intermingled male and female mice, we cannot determine whether their findings are consistent with our current findings.

**Figure 8.**
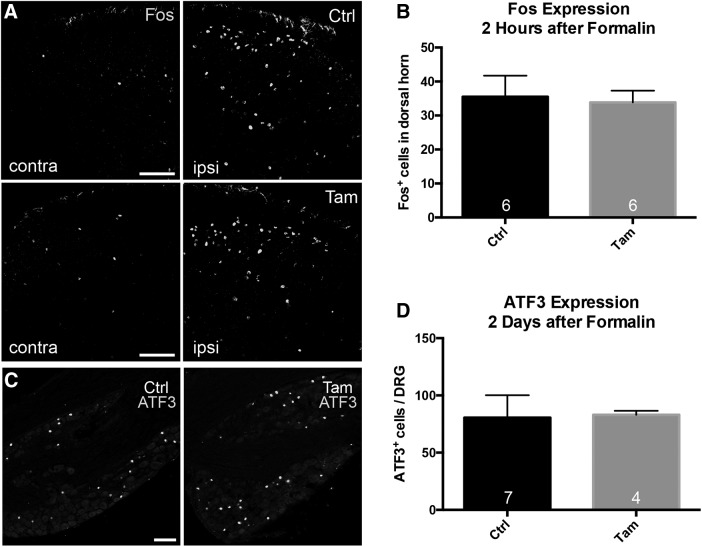
Formalin-induced expression of spinal cord Fos and primary afferent ATF3 in male BDNF-cKO mice. ***A***, ***B***, Two hours after formalin, control (Ctrl) and tamoxifen (Tam)-treated (cKO) mice showed a comparable increase of Fos immunoreactivity in the spinal dorsal horn ipsilateral to the injected paw. ***C***, ***D***, Both groups also showed equivalent ATF3 induction in L4/5 DRGs 2 d after the formalin injection, consistent with formalin producing peripheral axotomy. Data are mean ± SEM. Statistical significance was determined by Student’s *t* test. Numbers of animals are indicated on graphs. Scale bars = 50 μm (***A***) and 100 µm (***C***).

Some studies suggest that the second phase of the formalin test is a manifestation of central sensitization of dorsal horn nociceptive circuits that is driven by activity during the first phase (however, see Taylor et al., 1997; Shields et al., 2010). As central sensitization is also presumed to underlie post-formalin secondary hyperalgesia, in which there is mechanical hypersensitivity of intact regions near the inflamed hindpaw, we also examined post-formalin, mechanical thresholds in male animals, 1 d after the formalin injection (Zeitz et al., 2004). Figure 7*E* shows that, in fact, BDNF cKO mice displayed the same degree of mechanical hypersensitivity as control mice. In addition, spinal cord Fos expression, a marker of neuronal activity (Fig. 8*A*,*B*), and primary afferent expression of ATF3 (Fig. 8*C*,*D*), a marker of peripheral nerve injury, did not differ between control and BDNF cKO mice. We conclude that deletion of BDNF in the adult has limited influence on a variety of acute pain and itch behaviors.

### Primary afferent-derived BDNF does not contribute to persistent pain

**Figure 9.**
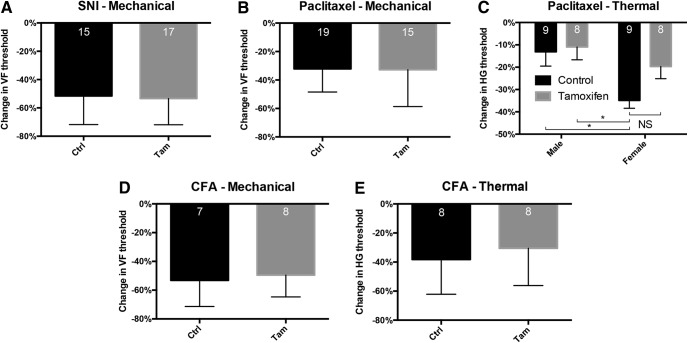
Tissue and nerve injury-induced hypersensitivity in BDNF-cKO mice. ***A***, Tamoxifen-treated cKO animals developed normal mechanical allodynia one week after SNI. ***B***, Mechanical allodynia also did not differ between tamoxifen- and corn oil-treated mice in a paclitaxel-induced chemotherapy model of neuropathic pain. ***C***, Unexpectedly, female control mice developed greater thermal hypersensitivity 12 d after paclitaxel compared to male groups. While this hypersensitivity decreased after tamoxifen-treatment, it did not do so significantly (*p* > 0.05). Mechanical allodynia (***D***) and thermal hypersensitivity (***E***) also did not differ between BDNF cKO and control mice 3 d after CFA injection. Data are mean ± SEM. For ***A***, ***B***, ***D***, ***E***, statistical significance was determined by Student’s *t* test. For ***C***, statistical significance was determined by two-way ANOVA with Bonferroni correction for multiple comparisons; *p < 0.05. Numbers of animals are indicated on graphs.

Here, we examined the consequence of deleting BDNF in various models of chronic neuropathic and inflammatory pain. We expected that the behavior of BDNF cKO animals would recapitulate previous findings that showed that inhibiting TrkB signaling reduces hyperalgesia and allodynia in multiple models of chronic pain (Fukuoka et al., 2001; Wang et al., 2009; Bao et al., 2014). We were surprised, therefore, to find that knock-out animals developed normal mechanical hypersensitivity in the SNI model of neuropathic pain (Fig. 9*A*). The hypersensitivity lasted throughout the 14-d test period. The knock-out and wild-type animals also developed comparable levels of mechanical hypersensitivity in the Paclitaxel model of chemotherapy-induced neuropathic pain (Fig. 9*B*,*C*). Finally, the BDNF cKO and control mice developed comparable mechanical and thermal (heat) hypersensitivity 3 d after injection of CFA (Fig. 9*D*,*E*), which generates a model of tissue injury-induced chronic inflammation.

## Discussion

In this study, we characterized the expression of primary afferent-derived BDNF, as well as its contribution to the processing of pain and itch-inducing messages. In distinct contrast to previous reports, we found that BDNF is expressed primarily by myelinated neurons, with a much smaller percentage of BDNF-positive neurons coexpressing the peptidergic marker CGRP. We next used a tamoxifen-inducible, primary afferent-specific Cre recombinase to delete BDNF selectively from adult, sensory neurons. This approach avoided the inevitable concern that because of its extensive expression in DRG and spinal cord during embryonic development, deletion of BDNF could produce changes that complicate the interpretation of BDNF phenotypes in the adult. With few exceptions, BDNF deletion did not alter pain- or itch-associated behaviors. We did observe that male knock-out animals were less responsive in the tail immersion and formalin tests, while the scratching induced by histamine was only affected (reduced) in female mice. Perhaps most surprisingly, despite ample evidence showing that BDNF expression changes dynamically in the setting of nerve injury (Cho et al., 1997; Zhou et al., 1999; Fukuoka et al., 2001; Obata et al., 2006; Lin et al., 2011; Cao et al., 2012), the BDNF knock-out animals developed normal mechanical and thermal hypersensitivity in several models of neuropathic and chronic inflammatory pain. Based on these findings, we conclude that sensory neuron-derived BDNF is not a necessary or significant contributor to acute pain or itch processing, or to the development of neuropathic or chronic inflammatory pain. Whether BDNF contributes to ongoing pain should be determined in future studies. Our findings contrast significantly with several previous reports (see below).

A major distinction between our anatomic analysis and previous studies of BDNF expression in sensory neurons is that we did not rely on BDNF antibodies. Because the BDNF null mutation is lethal (Ernfors et al., 1994), it is difficult to verify BDNF antibody. By using a Cre-dependent, knock-in reporter construct that drives β-Gal expression selectively in adult animals (Gorski et al., 2003), the analysis performed in the present report obviated this concern. Interestingly, although we found that diverse populations of sensory neurons express BDNF, myelinated afferents predominated, a finding at odds with earlier reports concluding that BDNF is expressed mainly in unmyelinated nociceptors (Michael et al., 1997; Thompson et al., 1999; Heppenstall and Lewin, 2001; Luo et al., 2001; Obata et al., 2006; Salio and Ferrini, 2016). Because TRPM8, peripherin, and NF200 largely label non-overlapping populations in the mouse (Dhaka et al., 2008; Takashima et al., 2010) and, together, account for the majority of DRG neurons, we conclude that our analysis characterized almost the entire population of BDNF neurons (NF200 = ∼67% overlap, peripherin = ∼12% overlap, and TRPM8 = ∼23% overlap).

Of particular note are the significant discrepancies between our results and those of Zhao et al. (2006), who deleted BDNF from sensory neurons using an NaV1.8-Cre cross. The authors reported hotplate hypersensitivity and formalin hyposensitivity in female mice after developmental BDNF deletion and no change in mechanical thresholds in a nerve injury induced model of neuropathic pain. Male mice were not tested in that study. However, when those authors selectively deleted BDNF in adult mice (Sikandar et al., 2018), they reported opposite results, namely decreased, responsiveness in the hot plate test and a reduction in mechanical hypersensitivity in a neuropathic pain model. Although the authors did not address these discrepancies, clearly the timing (adult vs embryonic) and extent of the BDNF deletion (partial in the Nav1.8-Cre cross vs complete in the Advillin-CreER cross) appears to be critical. Furthermore, in the NaV1.8 knock-out study, BDNF was not eliminated from all DRG neurons (Zhao et al., 2006), omitting some that have been implicated in mechanical allodynia after nerve injury (Seal et al., 2009; Abraira and Ginty, 2013). And, of course, compensatory molecular reorganization in the setting of developmental BDNF (NaV1.8-mediated) deletion could also have contributed to the differences in the observed phenotypes. Most surprising, however, is that using the same deletion (adult) protocol as (Sikandar et al., 2018), we only detected a significant effect of BDNF knock-out on the second phase of the formalin test, but in contrast to results from that report, we only observed the deficit in male mice.

In general, activity in the first phase of the formalin test is thought to drive dorsal horn central sensitization, which then catalyzes the second phase (Latremoliere and Woolf, 2009)^.^ Consistent with this view, BDNF increases the excitability of sensory neurons (Chen et al., 2014) and superficial dorsal horn neurons (Kerr et al., 1999; Garraway et al., 2003; Hildebrand et al., 2016) and leads to phosphorylation of spinal NMDA receptors (Slack and Thompson, 2002; Slack et al., 2004; Liu et al., 2015; however, see Pezet et al., 2002). These observations, when viewed in light of our results, suggest that BDNF may be necessary for spinal sensitization in the formalin model for male, but not female mice. Indeed, there is precedent for sexual dimorphism in BDNF signaling. For example, TrkB expression is regulated by androgens (Liu et al., 2012). As the formalin test is thought to model postsurgical pain, the sexual dimorphism we observed could have particular clinical relevance.

Overall, however, we found few pain or itch phenotypes after deleting BDNF selectively from primary afferent neurons. This is in stark contrast to numerous behavioral reports that found large behavioral effects after inhibiting BDNF/TrkB signaling, including studies from our own laboratory (Wang et al., 2009), where we used systemic pharmacological blockage of TrkB rather than selective sensory neuron deletion of BDNF. In that study we demonstrated that TrkB-signaling is necessary for behavioral sensitization after nerve injury, which agreed with many other studies that applied BDNF peripherally or administered intrathecal BDNF or TrkB scavengers (Shu et al., 1999; Thompson et al., 1999; Fukuoka et al., 2001; Groth and Aanonsen, 2002; Coull et al., 2005; Bao et al., 2014). Reconciling these findings with our new observations raises several possibilities. First, rather than these phenotypes deriving from sensory neurons, pain- and itch-relevant BDNF might originate in the spinal cord or from descending fibers from the brain (for example, see Hsieh et al., 2016). Importantly, in contrast with an earlier report that found no BDNF expression in neonatal mouse spinal cord (Heppenstall and Lewin, 2001), our *in situ* analysis in the adult revealed very extensive expression in spinal cord neurons. However, in agreement with a recent transcriptome analysis (Zhang et al., 2014), we could not demonstrate expression of BDNF in microglia, even in the fate map analysis. This result was surprising and suggests that the BDNF contribution to spinal cord processing of pain messages is, in fact, neuron derived. Second, NT-4/5, which also binds selectively to TrkB, might regulate aspects of pain processing widely attributed to BDNF. Third, conditional BDNF knock-out from sensory neurons, although generally considered more appropriate than embryonic deletion, could still produce compensatory changes that influence behavior. Nevertheless, its predominant expression in myelinated sensory neurons and limited contribution to pain and itch suggest that future studies should investigate primary afferent-derived BDNF as well as TrkB signaling in the context of low-threshold mechanotransduction.

## Synthesis

Reviewing Editor: Mary Heinricher, Oregon Health & Science University Decisions are customarily a result of the Reviewing Editor and the peer reviewers coming together and discussing their recommendations until a consensus is reached. When revisions are invited, a fact-based synthesis statement explaining their decision and outlining what is needed to prepare a revision will be listed below. Note: If this manuscript was transferred from JNeurosci and a decision was made to accept the manuscript without peer review, a brief statement to this effect will instead be what is listed below.

The overall findings here are twofold. The first is that BDNF is expressed in over half of the myelinated primary afferents examined (indicated by NF200), but that a non-trivial proportion of other neurons (CGRP+ most notably) also express BDNF. The second is that there is not an obvious change in pain behaviors, at least not anything consistent across assays.

Reviews for J. Neurosci. focused on a number of weaknesses. One was an apparent difference between the mice used for the anatomical vs. functional studies. Although the authors state in their rebuttal that the same mice were used for both, this would not be obvious from reading the manuscript itself.

Second were concerns as to validation of the deletion. The authors do argue convincingly that the assessment of message and protein is sufficient.

Third, reviewers queried why innocuous behaviors were not studied, since that is apparently the conclusion of the paper. Although this reviewer understands that anatomy was perhaps not known before the behavioral assays were performed, and would not require that this be done for publication in eNeuro, the authors should then have addressed the central issues raised by their behavioral data, considering factors that might contribute to discrepancies, and providing some argument for they consider their own study definitive.

Finally, there were concerns about statistical analyses. These concerns were not adequately addressed, and are discussed in more detail below.

Specific Comments

In general, the rationale for the studies described here is not well argued. The Statement of Significance, Introduction, and Discussion set up the core problem as being the inadequacy of BDNF antibodies, but the claimed ‘debates’ about these antibodies are never supported by a citation. More important, antibody specificity is not the issue for the functional studies, which comprise a significant proportion of the work presented. When previous functional findings are addressed, the consideration is superficial. A general consistency across the field when non-genetic manipulations were employed is downplayed, whereas inconsistencies among studies using genetic manipulations are not critically analyzed.

The abstract is a poor representation of the manuscript. The sentence beginning “In contrast to many previous reports...” is misleading, since prior work for the most part used different approaches (e.g., exogenous BDNF administration). The conclusion, that everyone should “focus on behaviors evoked by innocuous stimulation” sidesteps the central question, which is why this particular dataset should be considered definitive.

Methodological concerns.

A significant weakness relates to timing and the potential for plasticity with the genetic approach. This is difficult to evaluate because the protocol is not adequately described. We are told that that tamoxifen was injected over 5 days, and that tissue was taken for the ELISA two weeks after completing the tamoxifen protocol, but that information was not provided for any of the other variables examined. It nevertheless appears that behavioral testing was performed at a variety of time-points post-tamoxifen. This is critical, since plasticity triggered by modifications of BDNF might explain some of the variation across assays seen here, as well as some of the differences with the previous reports.

Statistical analysis is not high quality. The Methods section (p. 9) claims a within-subject design, but the Results report between-group comparisons. The approach is t-tests, but this is apparently done on the basis of an ‘eyeball’ of the data to determine whether there was an effect in either sex. If there appeared to be an effect in males or females, the two sexes were separated out with a t-test for each. Defining groups on the basis of the outcome and then doing a statistical test violates all of the underlying assumptions for means testing. A more appropriate approach would have been an ANOVA with sex and genotype as factors, and if no significant effect of sex, main effect of genotype could then be reported. If there was an effect of sex, a post-hoc test such as Holms-Sidak could have been performed within each sex.

In any case, insufficient information was provided to determine what tests were actually performed. Related to this, we might have expected 16 to 18 animals/group (stated 8 or 9 of each sex), a number of the tests have much larger n's than this, raising the question as to what the actual experimental unit was.

Minor points:

The formalin test is not a model of post-operative pain.

The authors should employ a grammar/spellchecker. Errors such as ‘BNDF’ are surprising in a resubmission manuscript.
